# Atrial Remodeling and Arrhythmia in Scimitar Syndrome: A Literature Review

**DOI:** 10.3390/jcdd13100507

**Published:** 2026-10-09

**Authors:** Abdullah Sahyouni, Ahmad Karzoun, Victor Gomez, Yannis Minetos, Sophia Pryor, Ha Quang Nhat Nguyen, Manasa Jaishankar, Arifa Akthar Niha, Nikolai Piskulich, Shreya Sreekanth, Robert Subbiondo

**Affiliations:** 1 Department of Internal Medicine, HCA Florida Blake Hospital, Morsani College of Medicine, University of South Florida (USF), Bradenton, FL 34209, USA; ahmad.karzoun@hcahealthcare.com (A.K.); victor.gomez@hcahealthcare.com (V.G.); robert.subbiondo@hcahealthcare.com (R.S.); 2 Department of Radiology Residents, The Ohio State University School of Medicine, Columbus, OH 43210, USA; yannis.minetos@osumc.edu; 3 Department of Chemistry, University of South Florida, Tampa, FL 33620, USA; pryor.sophia@outlook.com (S.P.); nguyenquangnhatha2005@gmail.com (H.Q.N.N.); manasa.jay.17@gmail.com (M.J.); arifa.akthar03@gmail.com (A.A.N.); anonymoushogigo@gmail.com (N.P.); sreekanthshreya@gmail.com (S.S.)

**Keywords:** arrhythmia, scimitar syndrome, congenital heart diseases, arrhythmia burden, atrial remodeling, intervention, electrophysiology, EP, cardiology, pediatric cardiology

## Abstract

Scimitar syndrome is a rare congenital form of partial anomalous pulmonary venous return characterized by right pulmonary venous drainage into the inferior vena cava, causing right-sided volume overload and increased risk of supraventricular arrhythmias, particularly in adolescents and adults. However, existing studies do not clearly describe reported arrhythmia patterns or how heart rhythm changes over time. This structured literature review aimed to characterize the reported atrial and supraventricular arrhythmias and conduction disturbances in patients with Scimitar syndrome and examine their relationship with atrial remodeling, hemodynamic burden, associated structural abnormalities, and surgical or catheter-based intervention. PubMed, Embase, Web of Science, and the Cochrane Library were searched from September 2026. Of 308 abstracts, 42 articles met the inclusion criteria (Scimitar syndrome patients, with broader PAPVR populations included as contextual evidence; literature reviews, abstract only publications, animal studies and non-English articles were excluded). Studies consistently showed that Scimitar syndrome is associated with right-sided volume overload linked to increased atrial and supraventricular arrhythmias, most commonly atrial fibrillation and flutter, with greater risk in patients with associated atrial septal defects or pulmonary hypertension. Because Scimitar syndrome can remain undiagnosed until adulthood, future studies should identify specific predictors of arrhythmia development to improve long-term electrophysiological outcomes.

## 1. Introduction and Objectives

Scimitar syndrome is a rare congenital cardiopulmonary anomaly characterized by partial anomalous pulmonary venous return (PAPVR) in which one or more right pulmonary veins drain anomalously into the inferior vena cava [1,2,3]. This aberrant venous connection results in a left-to-right shunt that produces chronic right-sided volume overload, progressive enlargement of right-sided cardiac chambers, and a spectrum of cardiopulmonary manifestations that may range from asymptomatic childhood presentation to significant adult morbidity [2,3]. The syndrome is frequently accompanied by additional structural abnormalities including atrial septal defects, right lung hypoplasia, and pulmonary hypertension, all of which further contribute to hemodynamic burden [4,5,6].

Chronic atrial stretch and remodeling are well-established substrates for the development of supraventricular arrhythmias [5,7,8]. Progressive right atrial dilation promotes fibrosis, conduction heterogeneity, and electrical instability, creating conditions favorable for atrial fibrillation, atrial flutter, and other supraventricular tachyarrhythmias [7,8,9]. Where in surgically treated patients, atrial incisions and postoperative scar formation may provide additional arrhythmogenic substrates through reentrant circuits or injury to sinus-node and atrial conduction pathways. These mechanisms have been extensively described in broader congenital heart disease populations; however, their specific manifestation in Scimitar syndrome remains incompletely characterized [9,10,11]. As survival into adulthood has become increasingly common among patients with congenital heart disease, arrhythmia burden has emerged as a major determinant of long-term outcomes, quality of life, and need for intervention [10,11,12].

Despite the recognized association between structural congenital heart disease and arrhythmogenesis, the literature describing arrhythmias in Scimitar syndrome is fragmented and largely limited to isolated case reports and small retrospective series [12,13,14]. Reported rhythm abnormalities include atrial tachycardia, atrial fibrillation, atrial flutter, and postoperative conduction disturbances; however, available studies provide inconsistent information regarding prevalence, age at onset, temporal progression, recurrence, and the distinction between pre-existing and de novo postoperative arrhythmias [13,14,15]. Furthermore, the effects of corrective surgery, surgical technique, catheter-based interventions, and long-term electrophysiologic outcomes remain uncertain [14,15]. Consequently, clinicians lack evidence-based guidance regarding optimal rhythm surveillance, timing of intervention, and risk stratification in this unique patient population [15,16].

A comprehensive synthesis of available data is therefore needed to better define the relationship between the anatomic features of Scimitar syndrome and the development of atrial arrhythmias. Systematic evaluation of published reports can help clarify which structural and clinical characteristics confer the greatest arrhythmic risk, how arrhythmia patterns evolve over time, and whether specific management strategies influence outcomes [15,16,17]. Establishing such a framework is particularly important as the population of adults with congenital heart disease continues to expand, and arrhythmias increasingly become a primary driver of morbidity [16,17,18,19].

The present literature review aims to consolidate and critically evaluate published evidence regarding atrial remodeling and supraventricular arrhythmias in patients with Scimitar syndrome. Specifically, the review examines reported arrhythmia phenotypes and their frequency when available; relationships with age, atrial remodeling, shunt burden, pulmonary hypertension, and associated congenital abnormalities; pre-existing, early postoperative, and late postoperative rhythm disturbances; rhythm outcomes following intervention; and limitations in the evidence relevant to long-term rhythm surveillance and management [17,18,19,20].

## 2. Methods

This study was conducted as a literature review, aiming to synthesize clinical evidence on atrial remodeling and arrhythmia burden in patients with Scimitar syndrome. A predefined protocol guided the search, selection, and extraction processes to ensure transparency, reproducibility, and methodological rigor. Electronic searches were conducted in PubMed, Embase, Web of Science, and the Cochrane library initially on 17 December 2025, and updated the search on 15 September 2026, to identify peer-reviewed clinical studies. The search strategy included terms related to both the underlying congenital anomaly and arrhythmic manifestations, such as “Scimitar syndrome,” “partial anomalous pulmonary venous return,” “atrial remodeling,” “atrial arrhythmia,” “supraventricular arrhythmia,” and related synonyms. Boolean operators (AND/OR) were applied to refine searches and ensure identification of studies addressing both structural atrial changes and arrhythmia outcomes. Searches were iteratively refined to maximize relevance while minimizing retrieval of irrelevant studies.

Studies were included if they reported human patients of any age with confirmed Scimitar syndrome and clinically relevant atrial arrhythmia, supraventricular arrhythmia, or conduction outcomes. Eligible study designs included randomized controlled trials, observational cohorts, case series, and case reports providing original clinical data, including patient demographics, associated cardiac anomalies, arrhythmia types, interventions performed, and rhythm outcomes. Exclusion criteria were narrative reviews, animal studies, abstracts without full-text availability, and non-English publications. Where Scimitar-specific evidence was insufficient to address postoperative conduction abnormalities, surgical technique, or rhythm-directed management, relevant studies involving broader partial anomalous pulmonary venous connection populations were considered separately as contextual evidence and were not used to calculate Scimitar-specific arrhythmia frequency.

Given the rarity of Scimitar syndrome and the limited number of studies reporting arrhythmic outcomes, relevant physiological concepts from related conditions, including PAPVR/PAPVC populations, left heart volume overload and atrial septal defects were also considered to contextualize findings. These concepts informed the interpretation of structural and electrophysiologic patterns but did not influence eligibility criteria or the structured search process. Inclusion and exclusion criteria are summarized in Table 1.

**Figure 1:**
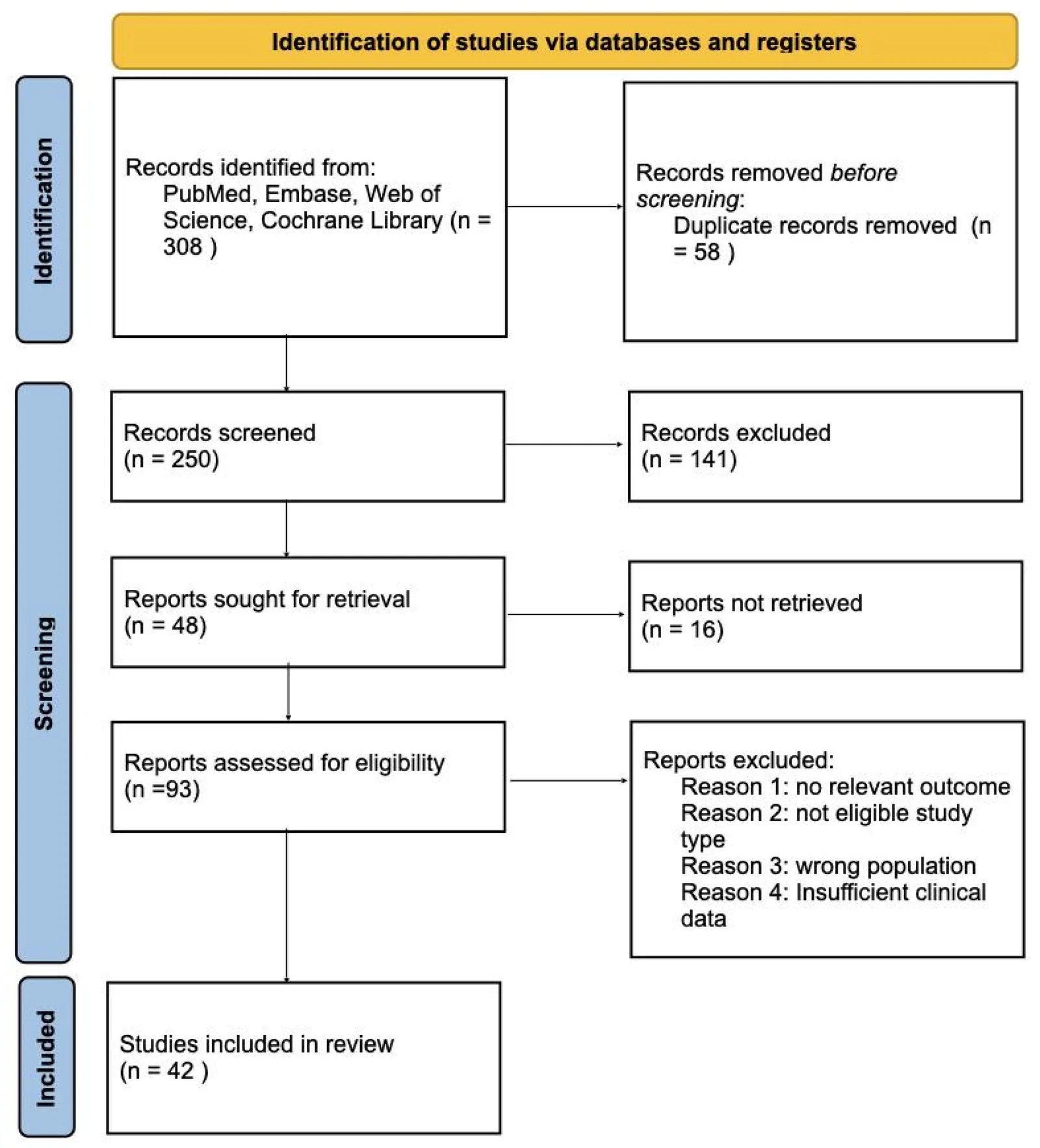
Diagram of study selection using PRISMA flow design.

The screening and selection process adhered to a two-stage review. Titles and abstracts of all retrieved articles were screened independently by five reviewers against predefined inclusion and exclusion criteria, and discrepancies were resolved by consensus. Of the 308 abstracts initially identified, 93 were selected for full-text review and 42 met the inclusion criteria. Reference lists of included studies were also manually screened to capture additional relevant publications. The overall selection process is depicted in the flow diagram in Figure 1.

Data extraction was conducted using a standardized form to ensure consistency and completeness. Identified limitations were documented and considered in interpretation rather than used as exclusion criteria, maintaining inclusivity while signaling methodological rigor. This approach provides a transparent and reproducible methodology while maintaining flexibility for the heterogeneity inherent to rare congenital cardiac conditions, enabling a comprehensive synthesis of structural and arrhythmic outcomes in Scimitar syndrome. Differences in study population, sample size, follow-up duration, rhythm-monitoring strategy, definitions of arrhythmia, and reporting of structural remodeling were considered when interpreting the evidence. Because of substantial clinical and methodological heterogeneity, findings were synthesized narratively rather than pooled quantitatively.

## 3. Results

### 3.1. Study Characteristics and Evidence Base

The included evidence was heterogeneous, consisting primarily of case reports and observational studies, with substantial variation in sample size, patient age, and follow-up. Hemodynamic measures, including Qp/Qs and pulmonary artery pressure, were inconsistently reported, while follow-up ranged from short postoperative assessments to several decades after repair.

Most rhythm-specific evidence was Scimitar-specific, while broader PAPVC studies were considered separately for contextual information on postoperative arrhythmias, sinus-node dysfunction, conduction abnormalities, and surgical technique. These broader findings were not used to determine Scimitar-specific arrhythmia frequency.

Study-level characteristics and reported arrhythmia outcomes from Scimitar syndrome and broader PAPVC/PAPVR studies directly discussed in the Results are summarized in Table 2.

### 3.2. Clinical Spectrum and Disease Expression

Clinical presentation ranges from severe infantile disease to delayed adult diagnosis [28,29,30,31,32]. This broad age distribution is relevant to arrhythmia evaluation because the duration of exposure to abnormal right-sided hemodynamic loading may vary substantially between patients [33,34]. In adults, rhythm abnormalities may become clinically apparent after years of otherwise unrecognized congenital disease and can contribute to the evaluation that ultimately identifies the underlying anomaly [21,35].

### 3.3. Arrhythmia Phenotypes and Frequency

Scimitar-specific reports described a heterogeneous spectrum of rhythm abnormalities, including atrial fibrillation, atrial flutter, ectopic atrial tachycardia, and atrioventricular conduction abnormalities [21,22,23,24]. In a pediatric cohort of 22 patients, postoperative no sustained ectopic atrial tachycardia occurred in 1 patient (4.5%) approximately 3 weeks after repair [22], whereas another pediatric cohort of 11 patients reported no perioperative arrhythmias [23]. In an adult case, incomplete right bundle branch block was documented before repair, with no reported rhythm complication during 3 months of follow-up [24]. Late rhythm disturbances have also been described; a 53-year-old patient developed paroxysmal atrial fibrillation followed by atrial flutter and intermittent second-degree atrioventricular block decades after repair [21].

Although right-sided chamber enlargement and pulmonary hypertension were commonly described [22,24,36], the included literature inconsistently reported longitudinal right-atrial volume, strain, or other quantitative remodeling measures alongside rhythm outcomes. Therefore, the available studies do not establish a specific right-atrial volume, strain, shunt magnitude, or other remodeling threshold associated with subsequent arrhythmia development [22,24,34].

Overall, arrhythmias were reported both shortly after surgical repair and many years later [21,22]. However, the limited Scimitar-specific rhythm evidence, heterogeneous follow-up, and inconsistent rhythm surveillance prevent calculation of a reliable Scimitar-specific prevalence or incidence or identification of a dominant arrhythmia phenotype. Findings from broader PAPVC/PAPVR populations were therefore considered separately as contextual evidence and were not used to estimate Scimitar-specific arrhythmia frequency.

### 3.4. Postoperative Arrhythmias, Sinus-Node Dysfunction, and Surgical Technique

Postoperative rhythm abnormalities included both atrial tachyarrhythmias and conduction disturbances, including sinus-node dysfunction and related brady arrhythmic manifestations [21,25,27,37,38]. Postoperative rhythm abnormalities may occur in the setting of pre-existing atrial remodeling, while surgical effects on atrial tissue or conduction pathways may provide an additional postoperative substrate [21,25]. However, Scimitar-specific studies inconsistently distinguished pre-existing rhythm disease from de novo postoperative abnormalities, limiting estimation of postoperative arrhythmia incidence specifically in Scimitar syndrome.

Broader PAPVC evidence provides additional information regarding the relationship between surgical technique and postoperative rhythm abnormalities. Buz et al. [25] evaluated 119 patients undergoing PAPVC repair, all of whom were in sinus rhythm before surgery. New-onset nodal rhythm and atrial dysrhythmias occurred in 26.5% of patients undergoing repair through a right atriotomy compared with 54.5% of patients in whom the incision extended through the cavoatrial junction (*p* < 0.004). At hospital discharge, dysrhythmias remained present in 14.0% and 32.7% of patients, respectively. Among 58 patients evaluated at 1-year follow-up, arrhythmias were present in 6.25% and 18.1% of the respective groups [25].

Because all patients in the Buz et al. cohort were in sinus rhythm before surgery, these abnormalities represented de novo postoperative rhythm disturbances rather than recurrence of documented preoperative arrhythmias [25]. The greater frequency associated with extension through the cavoatrial junction supports an association between more extensive atrial/cavoatrial incision and postoperative rhythm disturbance, including potential sinus-node or conduction-pathway injury [25]. However, because this study involved a broader PAPVC population, these frequencies should be interpreted as contextual surgical evidence rather than Scimitar-specific postoperative arrhythmia estimates.

### 3.5. Rhythm-Directed Management and Catheter Ablation

Management differed depending on the type of rhythm abnormality. In broader PAPVC populations, postoperative sinus-node dysfunction and bradyarrhythmia’s sometimes required permanent pacing [27,37]. These studies did not report outcomes specifically for patients with Scimitar syndrome, so their findings cannot be interpreted as Scimitar-specific management outcomes.

Data on catheter ablation were also largely limited to broader PAPVR populations. Demarchi et al. [26] reported outcomes in nine adults with PAPVR who underwent atrial fibrillation ablation with radiofrequency or cryoballoon pulmonary vein isolation. Anomalous pulmonary veins were isolated in selected patients. During a median follow-up of 40 months, atrial fibrillation recurred in 4 of 9 patients (44%), with recurrences at 4, 17, 24, and 25 months [26].

Catheter ablation has therefore been performed in patients with anomalous pulmonary venous anatomy, but the available evidence remains small. Most of the data come from broader PAPVC/PAPVR populations rather than Scimitar syndrome itself, making it difficult to determine the optimal ablation approach or Scimitar-specific recurrence rates.

### 3.6. Management of Scimitar Syndrome and Indications for Intervention

Management approaches vary significantly depending on symptom severity and hemodynamic impact, reflecting the heterogeneity of the condition [21,22,33].

Treatment strategies of symptomatic patients with small shunts include embolization of systemic collaterals and surgical redirection of pulmonary venous flow to the left atrium [22,39,40,41]. Asymptomatic patients with small shunts are often managed conservatively with surveillance [22,41,42].

The decision to intervene is primarily guided by shunt magnitude and hemodynamic burden, often quantified using Qp:Qs ratios and invasive measurements [43,44]. This reinforces the importance of comprehensive evaluation prior to treatment selection.

### 3.7. Long-Term Rhythm Outcomes and Prognosis

Prognosis varies widely and is strongly influenced by age at presentation, shunt magnitude, and the presence of pulmonary hypertension [21,28,32].

Long-term evidence suggests that both tachyarrhythmias and bradyarrhythmia’s may emerge years after surgical repair, although Scimitar-specific estimates remain limited. Nielsen and Hjortdal [27] evaluated 83 patients after PAPVC repair with postoperative follow-up extending to 46 years. New-onset atrial fibrillation or atrial flutter developed in 4 patients (5%), sinus-node disease was identified in 9 patients (11%), and permanent pacemaker implantation was required in 7 patients (8%), occurring at an average of 12.7 years after surgery [27]. Overall, 67 of 83 patients (81%) remained free of bradyarrhythmia’s, tachyarrhythmias, or pacemaker requirement during follow-up [27].

Scimitar-specific evidence also demonstrates the possibility of late rhythm disturbances after repair. Cacciapuoti et al. [21] described late atrial arrhythmias together with atrioventricular conduction disease in a patient with previously repaired Scimitar syndrome, illustrating that rhythm abnormalities may become clinically apparent long after surgical intervention. However, the broader PAPVC cohort data and limited Scimitar-specific reports do not permit calculation of a reliable long-term arrhythmia incidence specifically for Scimitar syndrome. Differences in follow-up duration and rhythm-surveillance methods further limit direct comparison across studies [21,27].

## 4. Discussion

The available literature supports a relationship between chronic right-sided hemodynamic loading, atrial remodeling, and arrhythmia development in Scimitar syndrome, but the strength and precision of this evidence are limited by substantial heterogeneity. Direct Scimitar-specific rhythm data are sparse, and studies vary in patient age, anatomy, intervention status, follow-up duration, rhythm-monitoring strategy, and definitions of arrhythmia. Consequently, the literature does not support a reliable, pooled estimate of arrhythmia prevalence or age-specific incidence [34,44].

Taken together, the findings support a mechanistic framework in which persistent volume loading may promote atrial dilation and structural remodeling, including fibrosis, with resulting electrical instability that may contribute to atrial fibrillation, atrial flutter, and related supraventricular tachyarrhythmias [21,25]. This trajectory mirrors remodeling-driven arrhythmogenesis described across other congenital heart disease populations, where rhythm disease increasingly contributes to long-term morbidity as patients survive into adolescence and adulthood [6,34,43]. The available studies do not establish a validated right-atrial volume, strain, or other remodeling threshold above which arrhythmia risk persists despite repair. Similarly, the literature does not identify a specific postoperative time point at which arrhythmic risk consistently peaks. These limitations prevent definition of a single intensified-monitoring interval from the current evidence.

**Figure 2:**
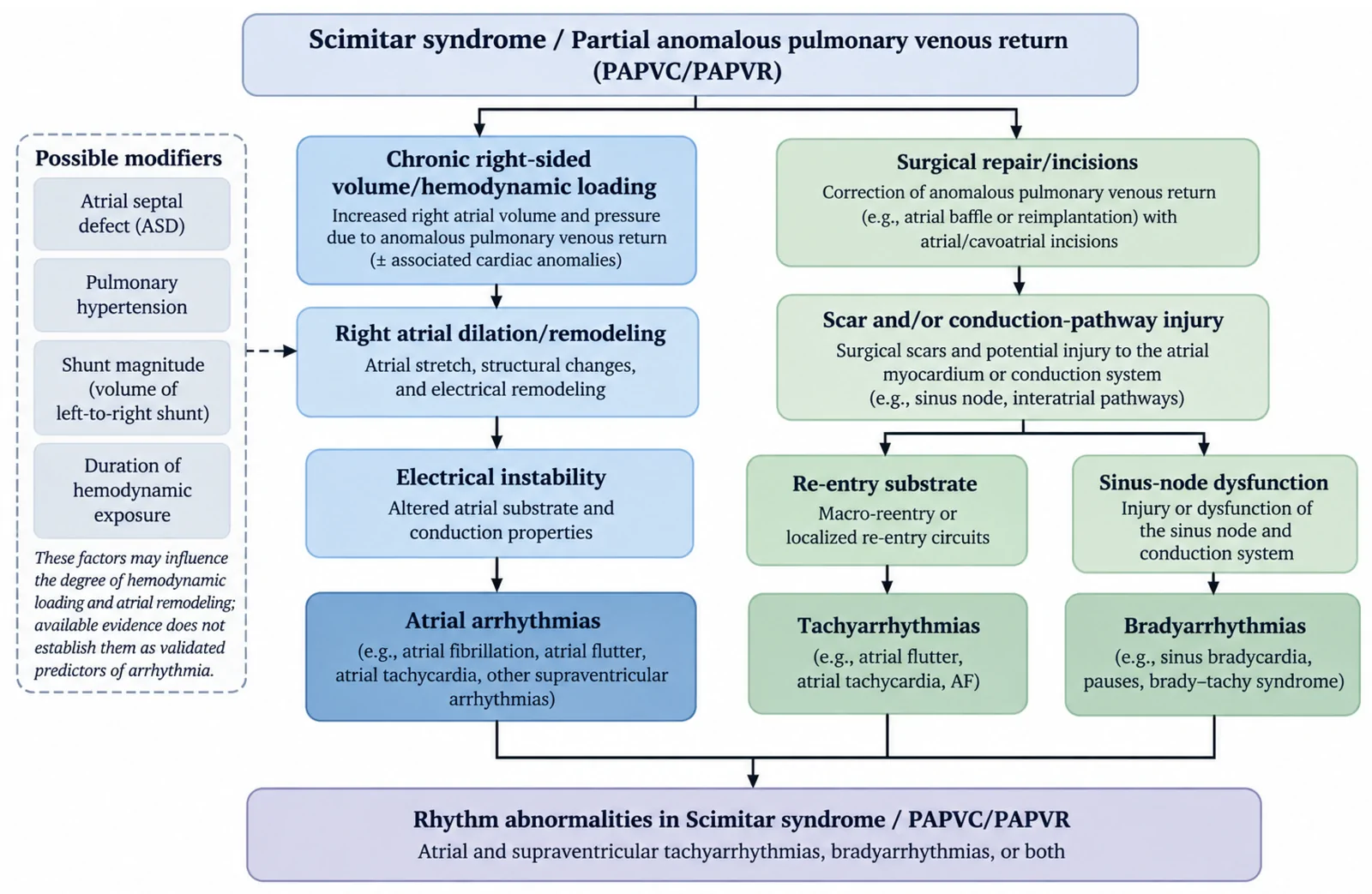
Proposed pathways of arrhythmia development in Scimitar syndrome and partial anomalous pulmonary venous connection/return (PAPVC/PAPVR).

The findings also suggest that preoperative and postoperative arrhythmogenic mechanisms should be considered separately. Before repair, this may come from years of right-sided volume loading and atrial remodeling, while after repair, surgical scars or injury to conduction pathways may create new sites for re-entry or sinus-node dysfunction [21,25]. These proposed hemodynamic and postoperative mechanisms of arrhythmia development are summarized in Figure 2.

Clinically, the evidence supports early and continued rhythm surveillance as part of routine follow up, particularly for adolescents and adults, given that arrhythmias may be intermittent and asymptomatic and can emerge after years of abnormal loading [21,43]. This has direct relevance for adult congenital heart disease care, where longitudinal electrophysiologic monitoring and timely detection of atrial arrhythmias are central to reducing downstream complications and guiding management [34]. In patients who develop atrial fibrillation or flutter, anticoagulation decisions also require consideration of thromboembolic and bleeding risk. The applicability and limitations of conventional CHA_2_DS_2_-VASc and HAS-BLED scoring in congenital heart disease should be considered alongside congenital anatomy, hemodynamic status, and other individual risk factors [45].

Several factors may affect how much right-sided loading occurs in patients with Scimitar syndrome. These include atrial septal defects, pulmonary hypertension, shunt magnitude, and how long the patient has been exposed to abnormal hemodynamics [34,36,46]. However, current studies have not established any of these factors as independent predictors of arrhythmias in Scimitar syndrome. For now, they are better considered possible modifiers rather than established risk factors.

Rhythm outcomes after intervention remain variable. Early postoperative atrial arrhythmias are described in surgical cohorts, while late onset arrhythmias are also reported years after repair or in medically managed patients with persistent shunting, suggesting that repair may reduce ongoing hemodynamic stress but does not necessarily eliminate the possibility of subsequent arrhythmia [21,23,25].

Interpretation of the available evidence is limited by the predominance of case reports and small retrospective series, variable patient age and anatomy, inconsistent rhythm-monitoring strategies, nonstandard definitions of remodeling and arrhythmia outcomes, and heterogeneous follow-up duration. Larger studies often focus primarily on anatomy, hemodynamics, or surgical outcomes rather than rhythm-specific endpoints. In addition, some evidence regarding surgical technique, sinus-node dysfunction, and rhythm management is derived from broader partial anomalous pulmonary venous connection populations rather than Scimitar syndrome alone. These limitations restrict direct comparisons between studies and prevent reliable, pooled estimates of Scimitar-specific arrhythmia prevalence or incidence.

Future studies should pair standardized longitudinal rhythm surveillance with quantitative measures of atrial size, volume, strain, and other remodeling markers. Reporting should distinguish pre-existing arrhythmia, de novo postoperative arrhythmia, recurrence of pre-existing arrhythmia, and sinus-node or other conduction disease. This approach would help determine whether specific remodeling measurements or postoperative time periods identify patients at greater risk for subsequent rhythm abnormalities.

## 5. Conclusions

In conclusion, available evidence supports an association between chronic right-sided hemodynamic loading, atrial remodeling, and arrhythmia development in Scimitar syndrome; however, the prevalence, age distribution, and temporal progression of rhythm abnormalities remain incompletely defined. Atrial and supraventricular arrhythmias, as well as sinus-node and other conduction abnormalities, have been reported both after repair and during long-term follow-up. Associated factors such as atrial septal defects and pulmonary hypertension may modify right-sided loading, although they have not been established as independent predictors of arrhythmia. From a clinical perspective, arrhythmias represent an under-characterized source of morbidity, and continued rhythm surveillance should be included in the long-term management of Scimitar syndrome. Proactive monitoring strategies may facilitate earlier detection and intervention, with potential implications for symptom control and overall outcomes.

## Figures and Tables

**Table 1:** Inclusion and exclusion criteria of the articles that underwent initial screening.

Inclusion Criteria	Exclusion Criteria
Human patients with confirmed Scimitar syndrome	Animal studies
Reported atrial arrhythmias, supraventricular arrhythmias, or any clinically relevant conduction disturbances	Narrative/literature reviews
Primary studies: case reports, case series, RCTs	Non-English publications
All age groups (Pediatrics and Adults)	Abstract-only publications
Publication identified from database inception to 15 September 2026	

**Table 2:** Selected studies reporting arrhythmia and conduction outcomes in Scimitar syndrome and broader partial anomalous pulmonary venous connection/return (PAPVC/PAPVR) populations.

Study	Population/Design	Key Findings	Timing	Management/Follow-Up	Evidence Type
Cacciapuoti et al., 2025 [21]	Case report; previously repaired Scimitar syndrome	Paroxysmal atrial fibrillation, atrial flutter, and intermittent second-degree atrioventricular block	Late; decades after repair	Medical therapy, cardioversion, and leadless pacemaker	Scimitar-specific
Geggel et al., 2020 [22]	Pediatric cohort; 22 patients	Sustained ectopic atrial tachycardia in 1/22 patients (4.5%)	About 3 weeks after repair	Postoperative follow-up after repair	Scimitar-specific
Cheng et al., 2021 [23]	Pediatric cohort; 11 patients	No perioperative arrhythmias reported	Perioperative	Surgical repair; postoperative follow-up	Scimitar-specific
Cruz-Galbán et al., 2021 [24]	Adult case report	Incomplete right bundle branch block before repair; no reported rhythm complication during follow-up	Preoperative; 3-month follow-up	Surgical repair	Scimitar-specific
Buz et al., 2009 [25]	Observational study; 119 PAPVC repairs; all in sinus rhythm preoperatively	New nodal rhythm/atrial dysrhythmias were more frequent when the incision extended through the Cavo atrial junction than with right atriotomy alone	Early postoperative, discharge, and 1 year	Surgical PAPVC repair	Broader PAPVC/PAPVR
Demarchi et al., 2022 [26]	Nine adults with PAPVR undergoing atrial fibrillation ablation	Atrial fibrillation recurred in 4/9 patients (44%)	Recurrences at 4, 17, 24, and 25 months	Radiofrequency or cryoballoon pulmonary vein isolation; median follow-up 40 months	Broader PAPVC/PAPVR
Nielsen & Hjortdal, 2021 [27]	Long-term cohort; 83 patients after PAPVC repair	New AF/flutter in 4/83 (5%); sinus-node disease in 9/83 (11%); pacemaker in 7/83 (8%)	Long-term postoperative follow-up, up to 46 years	Long-term rhythm follow-up after PAPVC repair	Broader PAPVC/PAPVR

## Data Availability

No new data were created or analyzed in this study. Data sharing is not applicable to this article.
